# Antibiotic Use in the First 2 Years of Life Is Related to Longitudinal BMI Trajectories From 0 to 18 years: A Prospective Cohort Study

**DOI:** 10.1155/jobe/9981660

**Published:** 2026-05-21

**Authors:** Jules J. Sing, Claire Gallagher, Xin Dai, N. Sabrina Idrose, Catherine M. Bennett, Michael J. Abramson, Shyamali C. Dharmage, Bircan Erbas, Caroline J. Lodge

**Affiliations:** ^1^ Allergy and Lung Health Unit, Melbourne School of Population and Global Health, The University of Melbourne, Carlton, Victoria, Australia, unimelb.edu.au; ^2^ Centre of Epidemiology and Biostatistics, Melbourne School of Population and Global Health, The University of Melbourne, Carlton, Victoria, Australia, unimelb.edu.au; ^3^ Institute for Health Transformation, Deakin University, Waurn Ponds, Australia, deakin.edu.au; ^4^ School of Public Health & Preventive Medicine, Monash University, Melbourne, Victoria, Australia, monash.ac.za; ^5^ School of Psychology and Public Health, La Trobe University, Melbourne, Victoria, Australia, latrobe.edu.au; ^6^ Centre for Food and Allergy Research, Murdoch Children’s Research Institute, Parkville, Victoria, Australia, mcri.edu.au

**Keywords:** allergy, antibiotics, cohort studies, obesity, overweight

## Abstract

**Background/Objective:**

Antibiotic exposure by 2 years is linked to increased childhood overweight/obesity risk, mainly in cross‐sectional studies. Few longitudinal studies exist in high‐risk populations, and none examined BMI trajectories up to 18 years. Our study is the first to investigate the association between early antibiotic exposure and BMI trajectories in a high allergy‐risk cohort.

**Methods:**

We analysed data from 428 participants in the Melbourne Atopy Cohort Study from birth to 18 years. Antibiotic use < 2 years was categorized as “ever versus never” and according to seven antibiotic classes. Number of courses (7 days) and the spectrum of antibiotics (broad versus narrow) were defined for each antibiotic class. Logistic regression models estimated associations between antibiotic use (any/specific), courses (0, 1, 2, and ≥ 3), and previously defined BMI trajectories from 4 weeks to 18 years: very low catch‐up, low average, stable average (reference), average increasing to very high (AIVH), and persistently high (PH).

**Results:**

We found consistent evidence of a relationship between antibiotic exposure by 2 years and increased risk of belonging to AIVH and PH trajectories, with magnitudes of effect differing by antibiotic spectrum and frequency. Notably, exposure to ≥ 3 courses of broad‐spectrum antibiotic was associated with the AIVH (aOR = 3.36 [95% CI: 1.03, 10.87]) and PH trajectories (aOR = 3.29 [95% CI: 1.03, 10.53]).

**Conclusion:**

These findings may have important clinical implications, but further large‐scale studies are required.

## 1. Introduction

Overweight and obesity are major public health issues worldwide. In Australia, they rank second for disease burden after tobacco use [1]. A suggested contributor to childhood and adolescent overweight and obesity is the disruption of gut microbiome, or dysbiosis [2], which may be related to antibiotic use in childhood [3]. Infants (< 2 years) are especially vulnerable to antibiotic‐related dysbiosis as their gut microbiome composition is highly unstable and dynamic [2]. A small case–control study nested in a large birth cohort (*n* = 1240) found that faecal samples of 60 antibiotic‐exposed infants had lower relative abundance of *Bifidobacterium* and higher abundance of *Faecalibacterium*, *Agathobacter*, and *Klebsiella* compared to 60 nonantibiotic‐exposed infants.

The relationship between antibiotic exposure and obesity has not been well‐explored with mid‐late adolescent BMI outcomes (12–18 years of age) largely unknown. A recent systematic review [4] suggested an association between antibiotics in early life (< 2 years of age) and higher risk of childhood/adolescence (2–12 years of age) overweight/obesity with a pooled estimate from 12 studies of RR = 1.14 [1.06–1.23]. However, this estimate had high heterogeneity (*I* ^2^ = 95.5%) with timing of antibiotic exposure varying widely (< 6 months to < 2 years of age). The ages at which BMI was measured also varied considerably. Most studies did not adequately adjust for confounders, and the quality of evidence (GRADE) for the pooled estimate was “very low” [4].

Broad spectrum and greater frequency of antibiotic exposure may both be related to increased risk of obesity, but evidence is inconsistent with variability between studies [4, 5]. Additionally, children with allergic disease may be particularly vulnerable as they may be exposed to greater amounts of antibiotics in early life; however, evidence remains inconclusive [6, 7].

Several factors could modify the relationship between infant antibiotic use and childhood overweight or obesity, including biological sex (higher in boys) [5, 6], exclusive breastfeeding up to 6 months (reduced risk) [8, 9], and infection [10, 11]. Infections are associated with alterations in the gut microbiota and may interact with antibiotic use to influence childhood BMI [2, 7, 11].

Only two previous retrospective cohort studies have explored longitudinal BMI trajectories with multiple measurements. They had inconsistent findings and were limited to 8 years of age [12, 13]. One study reported increased risk of overweight or obesity at 6 years of age for children exposed to antibiotics before age 2 years (aOR = 1.91 [1.20–3.03]) [12]. The second study found no difference in BMI at 8 years with similar antibiotic exposure timing (weight difference of −0.09 kg [−0.26, 0.08 kg] [13]. It is important to look at trajectories and not just the relationship at one time point because trajectories show how an individual’s BMI changes over time, helping to identify patterns and correlations with specific antibiotics [14]. This method may shed light on the potential long‐term effects of antibiotics on metabolism [14].

We aimed to investigate the relationship between antibiotic use in the first 2 years of life, including the type, spectrum and frequency, and longitudinal BMI trajectories over the whole of childhood and adolescence (0–18 years) in a prospective cohort at high allergic disease risk. We also planned to investigate potential modification by biological sex, exclusive breastfeeding, and infection type.

## 2. Participants and Methods

### 2.1. Study Design and Participants

We analysed data from the Melbourne Atopy Cohort Study (MACS), well described elsewhere [15]. Briefly, MACS began as a randomised control trial (RCT) of 620 infants (born 1990–94), investigating infant formulae and allergic disease risk, and has continued as an observational birth cohort [15], in line with previous practice [16, 17]. Participating children required at least one parent or older sibling with a history of self‐reported allergic disease [15].

Initial baseline assessment occurred just prior to birth. Telephone interviews collected data every 4 weeks after birth for the first 64 weeks, at 18 months, and 2 years [15]. Annual telephone surveys were conducted from ages 3–7 years [15]. At ages 12 and 18 years, probands participated in clinical examinations and completed questionnaires [15].

Approximately 93% of MACS participants were followed to 2 years [15]. Retention rates decreased to 78% at 6 years and 58% by 12 years [15], increasing to 68% at 18 years (434/620) [15]. Further details are available in the e‐Methods and Figure S1 in the online Supporting Information and the MACS cohort profile [15]. Briefly, children lost to follow‐up had a higher likelihood of younger parents who smoked and had lower educational attainment and SES compared to those retained. There were no differences found in terms of gender or familial history of allergic diseases, except for maternal hay fever. Mothers with hay fever had a 2.11‐fold [1.04–4.25] higher risk of dropout at 2 years [15].

### 2.2. Exposure: Antibiotics

Antibiotic use collected from each 4 weekly telephone interview (0–2 years) was classified into any exposure (ever versus never) and number of courses in the first 2 years of life (1 course = 7 days) [18, 19]. Antibiotic exposure was further subcategorised by class (and spectrum) and frequency as follows: no exposure (0 days), 1 course (7 days), 2 courses (14 days), and ≥ 3 courses (≥ 15 days). For spectrum, penicillins were classified as narrow‐spectrum antibiotics, which is supported the Australian Pharmaceutical Benefits Scheme (PBS) [20] (see Supporting Information Figure S2), clinical guidelines, and relevant studies [7, 10, 11]. Sulphonamides, cephalosporins, macrolides, and nitroimidazoles were categorized as broad spectrum [5, 7, 10].

### 2.3. Outcome: Body Mass Index (BMI) Trajectories

Prospectively collected anthropometric data, including weight and length/height, were obtained using measurement protocols across 25 time points: 18 follow‐ups from birth to 64 weeks; at 18, 21, and 24 months; 3–7 years; and at 12 and 18 years (clinical examinations). Weight (kg) and height (m) were self‐reported during prospective telephone interviews. These anthropometric measures were objectively collected by Mercy Hospital community nurses (0–2 years) and clinical nurses (12–18 years of age). Measurements between 3 and 7 years were parentally collected. BMI (kg/m^2^) was calculated using this information. Participants included in the present analysis had at least one BMI measurement in both infancy and adolescence (*n* = 434).

A previously established group‐based trajectory modelling (GBTM) analysis of BMI z‐scores from ages 0–18 years derived by standardizing to the 1990 British BMI Distribution for 428 MACS participants identified five distinct trajectories: “very low catch‐up (VLC)”, “low to average (LA)”, “stable average (SA)”, “average increasing to very high (AIVH)”, and “persistently high (PH)” [21]. This was used as our outcome measure. Details have been previously published [21]. The most complete dataset available at the time of analysis extended to 18 years of age. Further information is available in the e‐Methods (Supporting Information).

### 2.4. Participant Variables

Based on a directed acyclic graph (DAG; Supporting Figure S3), biological sex, exclusive breastfeeding, participant allergies, and common infant infections were identified as potential confounders. Biological sex was recorded at birth, and exclusive breastfeeding data were collected at four weekly intervals until 12 months of age and then dichotomized as ≤ 3 or > 3 months of exclusive breastfeeding [9]. Infant infections were ascertained from parental responses to 18 prospectively administered telephone questionnaires conducted by research nurses during the first two years of life, capturing episodes of upper respiratory tract infections (URTI), bronchitis, and otitis media (OM) [21]. Each infection type was categorised as 0, 1‐2, or ≥ 3 episodes, consistent with prior studies examining similar early‐life exposure windows (< 2 years) and BMI outcomes [7, 11]. Allergic diseases (asthma, eczema, and hay fever) were captured at the same time and via the ISAAC questionnaire at the 12‐ and 18‐year clinical exam follow‐ups [15].

### 2.5. Parent Variables

Formal education recorded at baseline was used as a proxy for parental socioeconomic status [7], creating a binary variable (“at least one parent with tertiary education” versus “neither parent with tertiary education”). Baseline smoking data were used to define “ever” versus “never” smoking for mothers and fathers (see e‐Methods).

### 2.6. Statistical Methods

Our analyses were conducted within a hypothesis‐testing framework to examine whether point estimates and associations were consistent across exposure categories and outcome groups. The emphasis was on evaluating the overall pattern and consistency of associations to minimize type 2 errors (false negatives), rather than on individual statistically significant results. Therefore, we considered that formal adjustment for multiple hypothesis testing was not required.

Collinearity of model covariates was assessed using pairwise correlations (pwcorr) and variance inflation factors (VIFs). Pairwise correlations were low (maximum correlation = 0.24), and all VIF values were ≤ 1.39, indicating no evidence of significant multicollinearity (Supporting Information Figure S4). The relationship between any antibiotic exposure (ever versus never) up to 2 years and BMI trajectories was examined using multinomial logistic regression (MLR), with the “SA” trajectory and “no antibiotic exposure” as reference groups. Six participants (428/434) were omitted due to incomplete antibiotic exposure data. The MLR was adjusted for biological sex, exclusive breastfeeding duration (≤ 3 or > 3 months), allergic conditions at 18 years (asthma, eczema, and hay fever), and common infections before 2 years (URTI, bronchitis, and OM), parental educational attainment, and maternal and paternal smoking (ever versus never).

Using MLRs, we also modelled relationships with BMI trajectories by antibiotic class, frequency (number of courses), and antibiotic spectrum. There was an insufficient sample size to include nitroimidazoles in individual class analyses.

We investigated effect modification by biological sex, exclusive breastfeeding, and infection type [2, 7]. Likelihood ratio tests (LRT) with a threshold of *p* ≤ 0.1 were used. If there was evidence of interaction, we performed a stratified analysis. All statistical analyses were conducted using Stata software (version 18; StataCorp, College Station, TX).

### 2.7. Ethics Approval

Ethical approval for the original MACS study was obtained from the Mercy Hospital Human Ethics Committee in 1988 (R88/06). Ethics approval for subsequent follow‐ups has been obtained from the Royal Children’s Hospital (REF: 28035 and 35179) and Melbourne University (REF 0715114). All participants provided written informed consent at each follow‐up.

### 2.8. Artificial Intelligence

AI was used as a companion to the editorial process for selected paragraphs throughout the manuscript. All authors edited for accuracy.

## 3. Results

### 3.1. Participant Characteristics by BMI Trajectories (Table 1)

Of the 428 participants with data on antibiotic exposure and BMI trajectories to 18 years, 76.1% reported ever having at least one allergic condition by 18 years. In early childhood (0–2 years), 91.1% of participants had experienced at least one episode of URTIs, 64.7% had had OM, and bronchitis was less common at 19.6%. Over half (55.3%) were exclusively breastfed for more than 3 months.

**TABLE 1:** Demographics across BMI trajectory groups for participants who had antibiotic exposure information (ages 0–18 years).

	Very low catch‐up (VLC) (*n* = 47, 11.0%)	Low to average (LA) (*n* = 146, 34.1%)	Stable average (SA) (*n* = 108, 25.2%) Base outcome	Average increasing to very high (AIVH) (*n* = 64, 15%)	Persistently high (PH) (*n* = 63, 14.7%)	*p* value
*Participant characteristics % (n); total sample size (n = 428)*						
Sex: female (*n* = 207)	57.5% (27)	47.6% (70)	45.5% (51)	43.8% (28)	48.4% (31)	0.660^a^
Exclusive breastfeeding^‡^						0.005^a^
≤ 3 months (*n* = 197)	48.9% (23)	40.8% (60)	35.7% (40)	62.5% (40)	53.1% (34)	
> 3 months (*n* = 237)	51.1% (24)	59.2% (87)	64.3% (72)	37.5% (24)	46.9% (30)	
Asthma ever: yes (*n* = 192)	36.2% (17)	49.7% (73)	39.3 (44)	48.4% (31)	42.2% (27)	0.323^a^
Hay fever ever: yes (*n* = 193)	44.7% (21)	49.0 (72)	36.6 (41)	42.2 (27)	50.0% (32)	0.293^a^
Eczema ever: yes (*n* = 235)	57.5% (27)	57.4% (84)	50.9% (57)	45.3 (29)	59.4% (38)	0.415^a^
Allergies ever: yes (*n* = 326) (Asthma, hay fever, and eczema)	27.7% (13)	17.7% (26)	31.3% (35)	28.1% (18)	25.0% (16)	0.133^a^

0‐2 years of age infection episodes*^‡^* (participants)						
Upper respiratory tract (URTI)^‡^						0.121^b^
Never (*n* = 41)	8.5% (4)	13.6% (20)	8.9% (10)	3.1% (2)	7.8% (5)	
1‐2 (*n* = 307)	68.1% (32)	70.8% (104)	68.8% (77)	82.8% (53)	64.1% (41)	
≥ 3 (*n* = 86)	23.4% (11)	15.7 (23)	22.3% (35)	14.1% (9)	28.1% (18)	
Bronchitis^‡^						0.807^b^
Never (*n* = 350)	83.0% (39)	80.3% (118)	78.6% (88)	81.3% (52)	82.8% (53)	
1‐2 (*n* = 64)	14.9% (7)	17.0% (25)	14.3% (16)	12.5% (8)	12.5% (8)	
3 (*n* = 20)	2.1% (1)	2.7% (4)	7.1% (8)	6.3% (4)	4.7% (3)	
Otitis media^‡^						0.078^a^
Never (*n* = 162)	38.3% (18)	44.2% (65)	33.9% (38)	34.4% (22)	29.7% (19)	
1‐2 (*n* = 122)	31.9% (15)	27.2% (40)	28.6% (34)	17.2% (11)	37.5% (45)	
≥ 3 (*n* = 155)	29.8% (14)	28.6% (42)	37.5% (42)	48.4% (31)	32.8% (21)	

*Parental Characteristics*						
Mother’s education^∗^						0.307^a^
Less than secondary or secondary education (*n* = 130)	37.5% (15)	36.8% (43)	28.9% (26)	46.3% (25)	40.4% (21)	
Tertiary education (*n* = 223)	62.5% (25)	63.8% (74)	71.1% (64)	53.7% (29)	59.6% (31)	
Father’s education^∗^						0.928^a^
Less than secondary or secondary education (*n* = 137)	42.5% (17)	37.6% (44)	36.7% (33)	38.8% (21)	43.1% (22)	
Tertiary education (*n* = 215)	57.5% (23)	62.4% (73)	63.3% (57)	61.1% (33)	56.9% (29)	
Parents’ education^∗^						0.304^a^
One or both parents’ tertiary education (*n* = 258)	72.5% (29)	75.2% (88)	78.9% (71)	68.5% (37)	63.5% (33)	
Mother’s smoking status^∗^						0.961^b^
Current or within 3/12 (*n* = 325)	4.3% (2)	5.5% (8)	7.2% (8)	7.8% (5)	6.3% (4)	
Former smoker (ceased within the past 6 months) (*n* = 80)	14.9% (7)	21.2% (31)	16.2% (18)	20.3% (13)	17.2% (11)	
Never (*n* = 27)	80.9% (38)	73.3% (107)	76.6% (85)	71.9% (46)	76.6% (49)	
Mother’s smoking (first 4 weeks)^‡^ Yes (*n* = 23)	2.4% (1)	2.8% (4)	9.7% (10)	6.5% (4)	6.7% (4)	0.176^b^
Father’s smoking status^∗^						0.221^a^
Current or within 3/12 (*n* = 290)	14.9% (7)	16.6% (24)	15.3% (17)	25.4% (16)	24.2% (15)	
Former smoker (ceased within the past 6 months) (*n* = 59)	17.0% (8)	13.1% (19)	9.9% (11)	20.6% (13)	12.9% (8)	
Never (*n* = 79)	68.1% (32)	70.4% (102)	74.8% (83)	54.0% (34)	62.9% (39)	
Father’s smoking (first 4 weeks)^‡^ Yes (*n* = 61)	12.2% (5)	12.0% (17)	15.5% (16)	19.4% (12)	18.3% (11)	0.601^a^
Mother ever smoked (preconception, in utero, and 4 weeks postpregnancy) Ever (*n* = 109)	19.2% (9)	26.7% (39)	25.2% (28)	28.1% (18)	23.4% (15)	0.829^a^
Father ever smoked (preconception, in utero, and 4 weeks postpregnancy) Ever (*n* = 140)	31.9% (15)	30.1% (44)	26.1% (29)	46.0% (29)	37.1% (23)	0.082^a^
Mother’s marital status*^∗^* % (n)						0.771^b^
Married or de facto (*n* = 424)	97.9% (46)	98.0% (144)	98.2% (110)	98.4% (63)	95.3% (61)	
Single or divorced (*n* = 10)	2.1% (1)	2.0% (3)	1.8% (2)	1.6% (1)	4.7% (3)	
Delivery mode^#^						0.295^a^
Vaginal birth (*n* = 191)	84.0% (21)	79.1% (68)	84.1% (58)	65.6% (21)	79.2% (23)	
Caesarean (elective and emergency) (*n* = 50)	16.0% (4)	20.9% (18)	15.9% (11)	34.4% (11)	20.7% (6)	

^∗^Measured at baseline.

^‡^Measured within the first 2 years of life.

^#^Measured at 18‐year follow‐up.

^a^ *p*‐value derived from chi‐square test.

^b^ *p*‐value derived from Fisher’s exact test.

The AIVH group had the highest proportion of children with 1‐2 episodes of URTI (82.8%) and the highest prevalence of ≥ 3 episodes of OM (48.4%). More participants had ≤ 3 months breastfeeding in the AIVH (62.5%) and PH (53.1%) groups, compared to the SA trajectory proportion at 35.7% (*p* = 0.005).

Participant characteristics for the whole sample are included in the online Supporting Information Table S1.

### 3.2. Parental Characteristics by BMI Trajectories

Overall, most participants had at least one tertiary educated parent (60.2%). Over a quarter of mothers (25.4%) and 32.7% of fathers reported ever smoking. Parents without tertiary education were most frequent in the AIVH (31.48%) and PH (36.7%) groups. Children in the AIVH group also had the highest rates of mothers (28.1%) and fathers (46.0%) who smoked.

### 3.3. Distribution of Antibiotic Exposure by 2 Years Across the BMI Trajectories (0–18 years) (Table 2)

Antibiotic exposure during the first 2 years of life was high, with 80.3% of participants receiving at least one course. Penicillins were the most common (69.3%), followed by any broad‐spectrum antibiotic (54.4%), most commonly cephalosporins (31.1%).

**TABLE 2:** Participant characteristic distribution of antibiotic use ≤ 2 years of age across BMI trajectory groups (0–18 years).

	Very low catch‐up (VLC) (*n* = 47, 11.0%)	Low to average (LA) (*n* = 146, 34.1%)	Stable average (SA) (*n* = 108, 25.2%) Base outcome	Average increasing to very high (AIVH) (*n* = 64, 15%)	Persistently high (PH) (*n* = 63, 14.7%)	*p* value
*Antibiotic use (n = 428)* ≤ *2 years of age* *^Þ^*, *^‡^* (%, number of participants who took each specific antibiotic)						
Any antibiotic (*n* = 354)	76.6% (36)	79.5% (116)	79.6% (86)	93.8% (60)	88.9% (56)	0.026^b^
Broad spectrum (sulphonamides, cephalosporins, macrolides, and nitroimidazoles) (*n* = 242)	48.9% (23)	48.6% (71)	52.8% (57)	64.1% (41)	65.1% (41)	0.094^a^
Penicillins (*n* = 297)	68.9% (32)	63.7% (93)	66.7% (72)	78.1% (50)	79.4% (50)	0.038^a^
Sulphonamides (*n* = 139)	27.7% (13)	21.2% (31)	19.4% (21)	29.7% (19)	41.3% (26)	0.015^a^
Cephalosporins (*n* = 176)	34.1% (16)	24.7% (36)	27.8% (30)	43.8% (28)	36.5% (23)	0.056^a^
Macrolides (*n* = 124)	8.5% (4)	17.8% (26)	23.1% (25)	25.0% (16)	28.6% (18)	0.063^b^
Nitroimidazoles (*n* = 23)	2.1% (1)	6.2% (9)	8.3% (9)	1.6% (1)	4.8% (3)	0.349^b^

*Note:* Total sample size (*N* = 428).

^Þ^Missing 6 participants.

^‡^Measured within the first 2 years of life.

^a^ *p*‐value derived from chi‐square test.

^b^ *p*‐value derived from Fisher’s exact test.

There was evidence of differential exposure across BMI trajectories for almost all antibiotic use measures (class and spectrum) (Table 2). For “any antibiotic” exposure, children in the AIVH and PH trajectories had the highest exposure rates (93.8% and 88.9%, respectively; *p* = 0.026).

Those belonging to the AIVH and PH trajectories also had higher exposure to penicillins (*p* = 0.038) and sulphonamides (*p* = 0.015), cephalosporins (*p* = 0.056), and macrolides (*p* = 0.063). The AIVH and PH trajectory groups were more exposed to broad‐spectrum antibiotics at 64.1% and 65.1% compared to reference group (SA) (52.8%). The only exception to this pattern was for nitroimidazoles, but these were reported in small numbers.

### 3.4. Associations Between Any Antibiotic Use up to 2 Years and BMI Trajectories (0–18 years) (Figure 1) (Supporting Table S2)

We found consistent evidence of a positive relationship between antibiotic use and BMI trajectories compared with the “never antibiotics” and the “SA” trajectories whilst accounting for confounders. For all five specific antibiotic categories with sufficient data (broad spectrum, penicillins, sulphonamides, cephalosporins, and macrolides), children exposed before 2 years of age were more likely to belong to AIVH or PH categories compared with the SA. A positive association was found for sulphonamide exposure and belonging to the PH trajectory (aOR = 3.69 [1.57–9.03]). There was also some evidence for a relationship with any antibiotic and AIVH (aOR = 5.19 [0.97–27.80]), penicillins with PH (aOR = 2.21 [0.79–5.65]), and cephalosporins with AIVH (aOR = 2.04 [0.88–4.73]).

**FIGURE 1:**
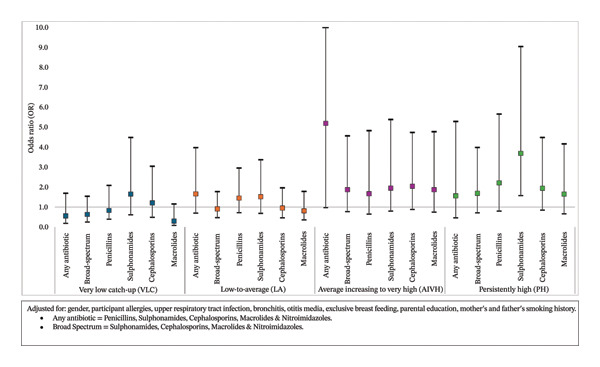
Multinomial logistic regression: adjusted odds ratios (95% CI)—antibiotic use ≤ 2 years of age across BMI trajectory groups (0–18 years). Adjusted for gender, participant allergies, upper respiratory tract infection, bronchitis, otitis media, exclusive breast feeding, parental education, and mother’s and father’s smoking history. (i) Any antibiotic = penicillins, sulphonamides, cephalosporins, macrolides, and nitroimidazoles. (ii) Broad spectrum = sulphonamides, cephalosporins, macrolides, and nitroimidazoles.

### 3.5. Associations Between Frequency of Antibiotic Use (by Class and Spectrum) and BMI Trajectories (0–18 years) (Table 3)

Infants exposed to 2 courses of any antibiotic before 2 years of age had a higher risk of following the AIVH trajectory (aOR = 11.15 [1.81, 68.51]). Additionally, exposure ≥ 3 antibiotic courses was associated with an elevated risk of belonging to the AIVH trajectory (aOR = 6.43 [1.01, 40.53]) and was modestly associated with the PH trajectory (aOR = 3.55 [0.89, 14.19]).

**TABLE 3:** Multinomial logistic regression: adjusted odds ratios (95% confidence intervals)—dose‐dependent antibiotic use ≤ 2 years of age across BMI trajectory groups (0–18 years).

	Very low catch‐up (VLC) (*n* = 47, 11.0%)	Low to average (LA) (*n* = 146, 34.1%)	Stable average (SA) (*n* = 108, 25.2%) Base outcome	Average increasing to very high (AIVH) (*n* = 64, 15%)	Persistently high (PH) (*n* = 63, 14.7%)
Antibiotic use in 2 years of age*^‡€^* (aOR, 95% CI, p value)					
*All types (n = 428)*					
Baseline (0 days) (*n* = 74)	(*n* = 11)	(*n* = 30)	(*n* = 22)	(*n* = 4)	(*n* = 7)
1–7 days (1 course) (*n* = 77)	0.54 (0.15, 1.91) *p* = 0.340 (*n* = 10)	1.21 (0.45, 3.26) *p* = 0.692 (*n* = 29)	(*n* = 21)	2.54 (0.39, 16.35) *p* = 0.327 (*n* = 9)	0.77 (0.18, 3.26) *p* = 0.722 (*n* = 8)
8–14 days (2 courses) (*n* = 94)	0.62 (0.17, 2.30) *p* = 0.471 (*n* = 10)	1.62 (0.57, 4.63) *p* = 0.371 (*n* = 25)	(*n* = 21)	11.15 (1.81, 68.51) *p* = 0.009 (*n* = 27)	1.35 (0.31, 5.89) *p* = 0.686 (*n* = 11)
≥ 15 days (≥ 3 courses) (*n* = 183)	0.57 (0.15, 2.17) *p* = 0.413 (*n* = 16)	2.65 (0.93, 7.50) *p* = 0.066 (*n* = 62)	(*n* = 44)	6.43 (1.01, 40.53) *p* = 0.048 (*n* = 24)	3.55 (0.89, 14.19) *p* = 0.073 (*n* = 37)

*Broad spectrum (n = 428) (sulphonamides, cephalosporins, macrolides, and nitroimidazoles)*					
Baseline (0 days) (*n* = 186)	(*n* = 23)	(*n* = 70)	(*n* = 49)	(*n* = 23)	(*n* = 21)
1–7 days (1 course) (*n* = 89)	0.42 (0.14, 1.22) *p* = 0.112 (*n* = 8)	0.52 (0.24, 1.28) *p* = 0.098 (*n* = 27)	(*n* = 29)	0.78 (0.27, 2.26) *p* = 0.640 (*n* = 11)	0.97 (0.36, 2.57) *p* = 0.945 (*n* = 14)
8–14 days (2 courses) (*n* = 69)	0.94 (0.24, 3.75) *p* = 0.935 (*n* = 7)	2.35 (0.85, 6.49) *p* = 0.099 (*n* = 28)	(*n* = 11)	3.67 (1.06, 12.65) *p* = 0.041 (*n* = 14)	2.20 (0.59, 8.10) *p* = 0.238 (*n* = 9)
≥ 15 days (≥ 3 courses) (*n* = 84)	1.15 (0.33, 4.08) *p* = 0.820 (*n* = 9)	1.14 (0.45, 3.39) *p* = 0.695 (*n* = 21)	(*n* = 19)	3.36 (1.03, 10.87) *p* = 0.043 (*n* = 16)	3.29 (1.03, 10.53) *p* = 0.044 (*n* = 19)

*Penicillins (n = 428)*					
Baseline 0 days (*n* = 131)	(*n* = 15)	(*n* = 53)	(*n* = 36)	(*n* = 14)	(*n* = 13)
1–7 days (1 course) (*n* = 79)	1.39 (0.45, 4.30) *p* = 0.562 (*n* = 13)	1.77 (0.70, 4.47) *p* = 0.228 (*n* = 26)	(*n* = 14)	2.10 (0.63, 7.00) *p* = 0.224 (*n* = 13)	2.46 (0.74, 8.15) *p* = 0.140 (*n* = 13)
8–14 days (2 courses) (*n* = 86)	0.61 (0.19, 2.03) *p* = 0.424 (*n* = 8)	1.10 (0.45, 2.67) *p* = 0.836 (*n* = 22)	(*n* = 23)	2.22 (0.72, 6.84) *p* = 0.162 (*n* = 21)	1.47 (0.43, 4.97) *p* = 0.535 (*n* = 12)
≥ 15 days (≥ 3 courses) (*n* = 132)	0.60 (0.18, 1.99) *p* = 0.406 (*n* = 11)	1.59 (0.67, 3.78) *p* = 0.296 (*n* = 45)	(*n* = 35)	1.03 (0.33, 3.29) *p* = 0.951 (*n* = 16)	2.46 (0.79, 7.69) *p* = 0.119 (*n* = 25)

*Sulphonamides (n = 428)*					
Baseline 0 days (*n* = 318)	(*n* = 34)	(*n* = 115)	(*n* = 87)	(*n* = 45)	(*n* = 37)
1–7 days (1 course) (*n* = 46)	1.19 (0.31, 4.60) *p* = 0.798 (*n* = 4)	1.16 (0.41, 3.35) *p* = 0.775 (*n* = 13)	(*n* = 10)	1.36 (0.39, 4.78) *p* = 0.628 (*n* = 7)	2.87 (0.92, 8.97) *p* = 0.070 (*n* = 12)
8–14 days (2 courses) (*n* = 26)	1.53 (0.22, 10.57) *p* = 0.661 (*n* = 3)	1.94 (0.43, 8.55) *p* = 0.382 (*n* = 10)	(*n* = 4)	2.20 (0.43, 11.05) *p* = 0.338 (*n* = 5)	3.77 (0.72, 19.62) *p* = 0.115 (*n* = 7)
≥ 15 days (≥ 3 courses) (*n* = 38)	2.81 (0.58, 12.58) *p* = 0.198 (*n* = 6)	1.92 (0.49, 7.45) *p* = 0.345 (*n* = 8)	(*n* = 7)	2.97 (0.69, 12.60) *p* = 0.140 (*n* = 7)	5.37 (1.29, 22.52) *p* = 0.022 (*n* = 10)

*Cephalosporins (n = 428)*					
Baseline 0 days (*n* = 295)	(*n* = 31)	(*n* = 110)	(*n* = 78)	(*n* = 36)	(*n* = 40)
1–7 days (1 course) (*n* = 54)	0.66 (0.18, 2.36) *p* = 0.525 (*n* = 6)	0.64 (0.25, 1.60) *p* = 0.341 (*n* = 15)	(*n* = 17)	0.83 (0.26, 2.67) *p* = 0.760 (*n* = 7)	1.32 (0.47, 3.71) *p* = 0.598 (*n* = 9)
8–14 days (2 courses) (*n* = 34)	3.96 (0.64, 24.39) *p* = 0.138 (*n* = 5)	3.01 (0.59, 15.30) *p* = 0.183 (*n* = 10)	(*n* = 3)	7.66 (1.44, 40.57) *p* = 0.017 (*n* = 11)	4.09 (0.67, 25.03) *p* = 0.127 (*n* = 5)
≥ 15 days (≥ 3 courses) (*n* = 45)	1.46 (0.36, 5.94) *p* = 0.600 (*n* = 5)	0.94 (0.30, 2.94) *p* = 0.917 (*n* = 11)	(*n* = 10)	2.73 (0.81, 9.56) *p* = 0.105 (*n* = 10)	2.57 (0.74, 8.89) *p* = 0.135 (*n* = 9)

*Macrolides (n = 428)*					
Baseline 0 days (*n* = 339)	(*n* = 43)	(*n* = 120)	(*n* = 83)	(*n* = 48)	(*n* = 45)
1–7 days (1 course) (*n* = 38)	0.54 (0.10, 2.95) *p* = 0.478 (*n* = 3)	0.72 (0.21, 2.46) *p* = 0.606 (*n* = 8)	(*n* = 8)	2.85 (0.78, 10.34) *p* = 0.112 (*n* = 8)	3.15 (0.95, 10.40) *p* = 0.060 (*n* = 11)
8–14 days (2 courses) (*n* = 31)	(*n* = 0)	1.01 (0.34, 3.51) *p* = 0.871 (*n* = 13)	(*n* = 9)	1.58 (0.37, 6.79) *p* = 0.535 (*n* = 4)	1.16 (0.25, 5.30) *p* = 0.846 (*n* = 5)
≥ 15 days (≥ 3 courses) (*n* = 20)	0.35 (0.03, 3.41) *p* = 0.365 (*n* = 1)	0.56 (0.13, 2.43) *p* = 0.437 (*n* = 5)	(*n* = 8)	1.11 (0.21, 5.85) *p* = 0.901 (*n* = 4)	0.38 (0.03, 3.82) *p* = 0.415 (*n* = 2)

*Note:* See “Supporting Information Figure S5” for graphical representation of Table 3.

^‡^Measured within the first 2 years of life.

^€^Adjusted for gender, participant allergies, upper respiratory tract infection, bronchitis, otitis media, exclusive breast feeding, parental education, and mother’s and father’s smoking history.

Infants exposed to two courses of broad‐spectrum antibiotics had a higher risk of following the AIVH trajectory (aOR = 3.67 [1.06, 12.65]). Exposure to ≥ 3 courses increased the risk for both the AIVH (aOR = 3.36 [1.03, 10.87]) and PH (aOR = 3.29 [1.03, 10.53]) trajectories. Analysis of individual broad‐spectrum antibiotic classes showed that two courses of sulphonamides were associated with a higher risk of belonging to the PH trajectory (aOR = 5.37 [1.29, 22.52]), while two courses of cephalosporins increased the risk of following the AIVH trajectory (aOR = 7.66 [1.44, 40.57]). Furthermore, exposure to macrolides (1, 2, or ≥ 3 courses) was generally associated with a higher risk of following the AIVH and PH trajectories but did not reach statistical significance.

For penicillin (predominately narrow spectrum), infants exposed to 1, 2, or ≥ 3 courses were overall more likely to belong to AIVH and PH groups when compared to the SA and no exposure to antibiotics.

Lastly, higher antibiotic consumption for any antibiotic, broad spectrum, sulphonamides, cephalosporins, and penicillin was generally associated with increased odds of belonging to some of the VLC and LA groups. However, these were not consistent nor statistically significant with many having wide confidence intervals that included the null.

## 4. Discussion

This is the first study to investigate the relationship between antibiotic use by type, class, spectrum, and frequency before the age of two years and longitudinal trajectories of BMI from birth to 18 years. Overall, we found that participants exposed to antibiotics within the first 2 years of life were associated with a higher risk of belonging to the AIVH and PH BMI trajectories compared to the SA trajectory.

Our novel use of BMI growth trajectories enhanced our ability to explore the association between early antibiotic exposure and childhood obesity [12, 13]. Our trajectories showed how an individual’s BMI changed over time, helping to identify patterns and correlations with specific antibiotics [12, 13]. Analysing BMI trajectories can lead to insights for assessing the potential association of antibiotic exposure and BMI growth, a critical aspect in paediatric care where monitoring development is essential [12, 13]. Furthermore, we used GBTM allowing us to differentiate between participants in the “AIVH” and “PH” BMI groups. In contrast, most previously published studies have classified participants only as either overweight or obese based on 1‐2 timepoints.

Our trajectory analysis found an increased risk for antibiotic exposure before the age of two and childhood‐adolescent overweight/obesity compared to previous systematic reviews. These reviews found adjusted pooled odds ratios (aORs) of 1.11 [1.02–1.20] and 1.14 [1.06–1.23]. However, differing from our approach, they combined overweight and obesity outcomes [3, 4]. Although these reviews analysed the same exposure window as our study, ages of BMI measurement varied widely. This resulted in significant heterogeneity of the pooled estimates (*I* ^2^ = 65.0% and 95.0%). A later prospective cohort [2] (*n* = 2140) found that infants exposed to antibiotics in the first year of life had increased risk of childhood overweight or obesity at 12 months (overweight: RR = 2.16 [1.53–3.06]; obesity RR = 2.93 [1.37–6.28]) and 2.5 years of age (overweight RR = 2.94 [1.91–4.53] and obesity RR = 3.07 [2.30–4.11]) [2]. However, they only included healthy mothers and infants who were exclusively breastfed for 6 months [2]. Previous studies have measured BMI at only 1‐2 timepoints limited to ≤ 12 years of age and did not use trajectories. Consequently, they have provided less information on changes in BMI over childhood (persistent or transitory) that can only be assessed in longitudinal studies.

Our prospective trajectory analysis examining antibiotic exposure by class, spectrum, and frequency is novel. Across most analyses, participants who received 2 courses or ≥3 courses of any antibiotic, broad‐spectrum antibiotics, penicillin sulphonamides, cephalosporins, or macrolides demonstrated increased odds of belonging to the AIVH or PH trajectories relative to the SA trajectory. Several of these associations were statistically significant. Regarding evidence for dose‐response, the sulphonamide AIVH trajectory as well as the any‐antibiotic, broad‐spectrum, and sulphonamide PH trajectories displayed increased odds with more courses—although confidence intervals varied considerably. Several antibiotic class analyses found the odds of belonging to the AIVH and PH groups were higher when exposed to two or three courses compared to one. Macrolides had an inverse relationship; however, our sample sizes were too small to make definitive conclusions. Previous macrolide studies have been contradictory, some finding increased risk of overweight or obesity, while others reported the opposite [22, 23].

A dose–response relationship has previously been found from meta‐analysis of three studies, for exposures by 24, 48, and 52 months and BMI up to 12 years of age [4]. A single antibiotic course was associated with obesity (RR = 1.08 [1.01–1.15]). However, 2‐3 courses yielded a RR of 1.22 [1.04–1.43] and ≥ 4 courses 1.31 [1.09–1.59] [4], although confidence intervals overlapped. In comparison, our OR point estimates were far higher for ≥ 3 courses of any antibiotic in the AIVH and PH groups (all types aOR range: 3.5–6.5). However, our confidence intervals were wider. This may be due to our smaller sample sizes with less precision or because the relationship between antibiotics and overweight/obesity may differ in high allergy‐risk children [6]. Of the MACS participants, 76.1% had either asthma, eczema, or hay fever, whereas the meta‐analysis studied children from the general population. Children with allergies may be more susceptible to the potential impact of antibiotics on BMI due to altered immune responses, concurrent medications (e.g., corticosteroids), and other lifestyle factors [6]. Whether this represents confounding, interaction, or a combination of both is not clear [6, 7]. We were unable to elucidate further in our analyses because of small numbers in the comparison group. Only one nested case‐control study has focused on asthma/allergy groups (asthma *n* = 246; control *n* = 477), investigating antibiotic use ≤ 12 months and BMI measurements at 9 and 12 years of age [6]. This study also found increased odds of childhood overweight in the asthma group compared to controls (aOR: 2.56 [1.36–4.79]) [6], providing some evidence that high allergy risk may be driving the increased risks seen in our study.

Our trajectory analysis found different relationships for broad and narrow‐spectrum antibiotics which may relate to differences in pharmacokinetics [5]. We found that exposure to broad‐spectrum antibiotics could pose a greater risk of subsequent childhood overweight and obesity than narrow‐spectrum antibiotics. Children exposed to 2 and ≥ 3 courses of broad‐spectrum antibiotics appeared to be at higher risk of following AIVH and PH trajectories than for the equivalent courses of narrow‐spectrum antibiotics. A meta‐analysis [4] investigating antibiotic exposure before 24 months and overweight/obesity between 2 and 10 years of age (mean 4.5 years) also found a slightly stronger relationship with broad‐spectrum antibiotics from 7 studies (RR = 1.10 [1.05–1.15]) than narrow‐spectrum antibiotics from 3 studies (RR = 1.01 [1.00–1.03]) [4]. However, the authors stated there were substantial methodological differences across included studies [4]. A prospective study [23] conducted after this meta‐analysis [21] found exposure to ≥ 2 courses of macrolides and cephalosporins (broad‐spectrum antibiotics) before 48 months was associated with a similar increased risk of obesity at 54 months to those who had been exposed to 4–6 courses of penicillins (≥ 2 macrolide courses aOR = 1.61 [1.18–2.21]; ≥ 2 cephalosporin courses aOR = 1.24 [0.90–1.70]; 4–6 penicillin courses aOR = 1.34 [0.71–2.50]). In contrast, a cross‐sectional study [24] showed minimal differences for risk of obesity at 4–5 years of age with a similar exposure window of 24 months (narrow‐spectrum aOR = 1.05 [1.03–1.07]; broad spectrum aOR = 1.03 [1.02–1.03]). No previous antibiotic spectrum studies modelled longitudinal trajectories.

Our asthma–allergy cohort limits generalizability of the findings. Many studies have examined general populations; therefore, this study provided an opportunity to investigate risks in an asthma–allergy cohort.

In addition to the advantage of using prospectively collected data for longitudinal trajectories (strengthening temporal relationships), the MACS provided a unique opportunity to examine antibiotic exposure during a period of high community antibiotic use. Collection of extensive prospective information allowed accurate and detailed information on important confounders (e.g., infant infections, socioeconomic status, breastfeeding, and maternal and paternal smoking) [7]. However, information on completion of each antibiotic course was not collected.

We were unable to independently assess the relationship with food allergies. However, all participants with food allergies were captured in our any‐allergy (asthma, eczema, and hay fever) variable.

Missing information at 18 years was predominantly from single mothers, those from lower socioeconomic backgrounds, and those with mothers who had hay fever or were smokers. However, it is unlikely that attrition at 18 years was related to antibiotic use during early life. For nondifferential bias, we could infer an underestimation of the true association between antibiotic exposure and BMI outcomes. If attrition was differential, it is not possible to determine which direction the results will change. Although some BMI data were missing, the GBTM approach is robust to missing data [21]. It can still identify similar BMI growth patterns in different groups (e.g. AIVH versus PH) over time [21].

We were unable to model linear associations because antibiotic exposure variables were highly skewed and nonlinear and statistical transformations did not improve normality. We also could not model closely spaced or recurrent antibiotic treatment that may cause greater disruption to gut microbiota, potentially leading to more pronounced effects on weight development [14].

Although MACS collected data frequently in childhood, it is possible that BMI increases may have preceded antibiotic use. However, reverse causation is unlikely as antibiotics were typically given after common infant infections [7, 10], and changes in BMI typically occur gradually over longer time periods [2]. Infant infections may themselves be linked with increased BMI, potentially causing confounding by indication [11]. A retrospective registry study (*n* = 260,556) using mixed effects logistic regression found infection alone may increase obesity risk in childhood and adolescence [11]. However, their comparison group (infants with infections, not treated with antibiotics) likely differed on several factors to the infants treated with antibiotics. Furthermore, it is the only study to date which found this association.

We were unable to adjust for prenatal and perinatal antibiotic exposure as well as parental BMI because this information was unavailable. A systematic review of 23 observational studies comprising 1,253,035 participants reported increased risk of overweight or obesity in a subgroup analysis for second‐trimester exposure (RR = 1.13 [1.06–1.22]; *p* = 0.001), but not when results were pooled across all trimesters [4]. Regarding maternal and paternal BMI, there is some evidence that these may influence risk of childhood‐adolescent overweight and obesity [25, 26].

Data limitations prevented examination of shorter age intervals (e.g., < 6 months) within the first 2 years of life. Systematic reviews have reported stronger associations with earlier exposure, particularly within the first 6 months of life (excluding the first 14 days) [3, 4, 27, 28]. For the first 14 days of life, two studies reported associations between antibiotic exposure and underweight at 12 months and 2 and 6 years of age, reflected in lower weight (kg) and BMI z‐scores (Z‐BMI) [29, 30]. Recent research indicates that antibiotic exposure may not significantly increase the risk of overweight/obesity after age two and we did not analyse this period [14].

Lastly, the relatively small sample size (*n* = 428) may have limited power and reduced our ability to fully explore associations involving less commonly used antibiotics.

## 5. Conclusion

Children exposed to antibiotics in the first 2 years of life in a high‐risk asthma–allergy cohort were more likely to belong to AIVH or PH trajectories in later childhood and adolescence (up to 18 years) than those not exposed. This risk was greater for those exposed to increased amounts of broad‐spectrum antibiotics. These findings highlight the need for clinicians to consider adhering to guidelines recommending judicious use of antibiotics, especially broad‐spectrum antibiotics. Larger studies are needed to corroborate these findings.

## Funding

The first 6 years of the MACS were funded (study formula and staff) by Nestec Ltd., a subsidiary of Nestlé Australia. The 12‐year follow‐up was funded by a project grant from the Asthma Foundation of Victoria. The National Health and Medical Research Council of Australia funded the 18‐year (APP454856) and 25‐year (APP1079668) follow‐up studies.

Open access publishing facilitated by The University of Melbourne, as part of the Wiley ‐ The University of Melbourne agreement via the Council of Australasian University Librarians.

## Disclosure

The named funders had no role in study design, data collection and analysis, decision to publish, or preparation of the manuscript. The funders had no impact on the outcome of the clinical reports collection. All bodies that have funded aspects of the MACS have had no role in the interpretation or publication of study findings.

## Conflicts of Interest

Professor Shyamali C. Dharmage and Caroline J. Lodge have received an investigator‐initiated grant from GSK and a partnership grant from AstraZeneca for unrelated research. Professor Michael J. Abramson has received investigator‐initiated grants from Pfizer, Boehringer‐Ingelheim, GlaxoSmithKline (GSK), and Sanofi for unrelated research. He has undertaken an unrelated consultancy for Sanofi and received a speaker’s fee from GSK. All of these grants were not related to this study or the current research paper. The other authors declare no conflicts of interest.

## Supporting Information

Additional supporting information can be found online in the Supporting Information section.

## Supporting information


**Supporting Information** E‐Methods: summary of MACS attrition, detailed descriptions of the derivation of maternal and paternal smoking variables, and additional information on the GBTM trajectory modelling. Supporting Figure S1: MACS cohort retention rates and data collection summary. Supporting Figure S2: Australian PBS define daily dose (DDD) rates (1990–1997) for penicillins and macrolides. Supporting Figure S3: directed acyclic graph. Supporting Figure S4: Stata pwcorr statistics—antibiotic exposure (days). Supporting Figure S5: graphs: multinomial logistic regression: adjusted odds ratios (95% confidence intervals)— dose‐dependent antibiotic use ≤ 2 years of age across BMI trajectory groups (0–18 years). Table S1: baseline demographics of the MACS cohort by BMI trajectory only (0–18 years). Table S2: demographics across BMI trajectory groups (ages 0–18 years) including additional smoking variables for participants who had antibiotic exposure information. Table S3: multinomial logistic regression adjusted odds ratios (95% CI)—antibiotic use ≤ 2 years of age across BMI trajectory groups (0–18 years). Table S4: sensitivity analysis: multinomial logistic regression: participant confounder effect size odds ratios (95% CIs)—all antibiotic type exposure variables ≤ 2 years of age across BMI trajectories (age 0–18 years). Table S5: sensitivity analysis: multinomial logistic regression: parental confounder effect size odds ratios (95% CI)—all antibiotic type exposure variables ≤ 2 years of age across BMI trajectories (age 0–18 years).

## Data Availability

Data are available on request depending on privacy/ethical restrictions.
