# Supplementary material for: Antibiotic Use in the First 2 Years of Life Is Related to Longitudinal BMI Trajectories From 0 to 18 years: A Prospective Cohort Study
Source: J Obes. 2026 May 21;2026:9981660. doi: 10.1155/jobe/9981660 (PMC13192243; doi:10.1155/jobe/9981660)
Supplement: Supplementary file 1 — Supporting Information E‐Methods: summary of MACS attrition, detailed descriptions of the derivation of maternal and paternal smoking variables, and additional information on the GBTM trajectory modelling. Supporting Figure S1: MACS cohort retention rates and data collection summary. Supporting Figure S2: Australian PBS define daily dose (DDD) rates (1990–1997) for penicillins and macrolides. Supporting Figure S3: directed acyclic graph. Supporting Figure S4: Stata pwcorr statistics—antibiotic exposure (days). Supporting Figure S5: graphs: multinomial logistic regression: adjusted odds ratios (95% confidence intervals)— dose‐dependent antibiotic use ≤ 2 years of age across BMI trajectory groups (0–18 years). Table S1: baseline demographics of the MACS cohort by BMI trajectory only (0–18 years). Table S2: demographics across BMI trajectory groups (ages 0–18 years) including additional smoking variables for participants who had antibiotic exposure information. Table S3: multinomial logistic regression adjusted odds ratios (95% CI)—antibiotic use ≤ 2 years of age across BMI trajectory groups (0–18 years). Table S4: sensitivity analysis: multinomial logistic regression: participant confounder effect size odds ratios (95% CIs)—all antibiotic type exposure variables ≤ 2 years of age across BMI trajectories (age 0–18 years). Table S5: sensitivity analysis: multinomial logistic regression: parental confounder effect size odds ratios (95% CI)—all antibiotic type exposure variables ≤ 2 years of age across BMI trajectories (age 0–18 years). [file JOBE-2026-9981660-s001.docx]

**Relationship Between Antibiotic Use in The First 2 Years of Life and Longitudinal BMI Trajectories From 0-18 Years: a Prospective Cohort Study**

Jules Sing^1,2^, Claire Gallagher^2^, Xin Dai^1^, N. Sabrina Idrose^1^, Catherine M. Bennett^3^, Michael J. Abramson^4^, Shyamali Dharmage^1^, *Bircan Erbas^5^, *Caroline Lodge^1,6^

Corresponding Author: Shyamali Dharmage

*^1^ Allergy and Lung Health Unit, Melbourne School of Population and Global Health, The University of Melbourne, Carlton, Victoria, Australia*

*^2^ Centre of Epidemiology and Biostatistics, Melbourne School of Population and Global Health, The University of Melbourne, Carlton, Victoria, Australia*

*^3^*Institute for Health Transformation, Deakin University, Waurn Ponds, Australia.

*^4^School of Public Health & Preventive Medicine, Monash University, Melbourne, Vic, Australia*

*^5^School of Psychology and Public Health, La Trobe University, Melbourne, Victoria, Australia.*

*^6^Centre for Food and Allergy Research, Murdoch Children's Research Institute, Parkville, Victoria, Australia*

**Denotes Equal Senior Author*

***E-methods*…………………………………………………………3**

***Supplementary Figure S1………………………………………..4***

***Supplementary Figure S2 ………………………………………..5***

***Supplementary Figure S3 ………………………………………..6
Supplementary Figure S4 ………………………………………..7
Supplementary Figure S4 ………………………………………..8
References*………………………………………………………….9**

***Table S1*……………………………………………………….......10**

***Table S2*……………………………………………………….......11**

***Table S3*……………………………………………………….......12**

***Table S4*……………………………………………………….......14**

***Table S5*……………………………………………………….......16**

**E-Methods**

**MACS Attrition Summary**^[1]^

In the MACS prospective study, there has been little evidence of selective participation attrition within the MACS. Children lost to follow-up were more likely to have younger and less educated parents compared to those who remained in the cohort. Furthermore, children lost to follow-up by age 2 were more likely to have mothers with self-reported hay fever. At both 2 and 18 years, children of parents who smoked had higher rates of being lost to follow-up. Additionally, children whose parents were renting their home and whose father's occupation was linked to lower socioeconomic status were more likely to be lost. No significant differences were observed between children lost to follow-up and those retained in terms of gender, family history of other allergic diseases (excluding maternal hay fever), early signs of atopy or eczema in the child, or various residential characteristics such as pet ownership.

**Derivation of the Group Based Trajectory Method for BMI (Outcome Measure)**

Prospectively collected anthropometric data, including weight and length/height, were obtained using measurement protocols across 25 timepoints: 18 follow-ups from birth to 64 weeks; at 18, 21, and 24 month, 3-7 years and at 12 and 18 years (clinical examinations). Weight (kg) and height (m) were self-reported during prospective telephone interviews. These anthropometric measures were objectively collected by Mercy hospital community nurses (0-2 years) and clinical nurses (12-18 years-of-age). Measurements between 3-7 years were parentally collected. Body mass index (BMI; kg/m²) was calculated using this information. Participants included in the present analysis had at least one BMI measurement in both infancy and adolescence (n = 434))^[2]^. Most participants (n=315) had at least 1 measure of BMI during infancy (0-2 years; 12 measures on average), 1 during childhood (3-7 years; 1.5 measures on average) and 1 during adolescence (18 years; 1 on average). While 119 participants had at least 1 measure of BMI during infancy and childhood, but not at 18 years. Both groups contributed to the models.

A Group Based Trajectory Modelling (GBTM) of BMI z-scores from ages 0-18 years was derived in a pervious study^[2]^ by standardising to the 1990 British BMI Distribution for 428 MACS participants and identified five distinct trajectories: 'Very-Low-Catch-Up (VLC)', 'Low-to-Average (LA)', 'Stable Average (SA)', 'Average-Increasing-to-Very-High (AIVH)', and 'Persistently High (PH)'^[2]^. The details on how these trajectories were derived are provided in an associated study^[2]^. In brief, BMI trajectories were derived using group-based trajectory modelling (GBTM) in Stata (Jones & Nagin, 2007), applying a censored normal model with age in years as the time variable. A stepwise approach was used to determine the optimal number and shape of trajectories^[2]^. Models with two to six classes were fitted, sequentially reducing polynomial order until all terms were significant^[2]^. Model selection was guided by the lowest Bayesian (BIC) and Akaike (AIC) Information Criteria, average posterior probability (APP) > 0.80, ≥ 5% of participants per class, and interpretability of trajectory shapes^[2]^. The five-class model was selected as optimal, showing the best fit (lowest BIC and AIC), high classification accuracy (APP = 0.89), and distinct, interpretable trajectories. Although data collection for the MACS cohort continued until 25 years-of-age, the most complete dataset available at the time of our analysis extended to 18 years-of-age^[2]^”

**Maternal and Paternal Smoking Variables**

Smoking data was collected at baseline and at 4-weeks post-partum. Parents were asked whether they were: a) smoking now, b) not smoking but had in past 3-months, c) not smoking now but had in the past 6-months, d) had smoked greater than 6/12 ago and e) had never smoked”. Frequency of smoking data was also collected for the first 4-weeks post-pregnancy. Parents were asked to tick one of the following boxes: "No cigarettes, smokes 1-4, 5-10, 11-20 or more than 21 cigarettes per day". The smoking data was then then categorised into "Ever" or “Never” for paternal and maternal^[3]^.

**Supplementary Figure S1: MACS Cohort Retention Rates & Data Collection Summary** ^[1]^


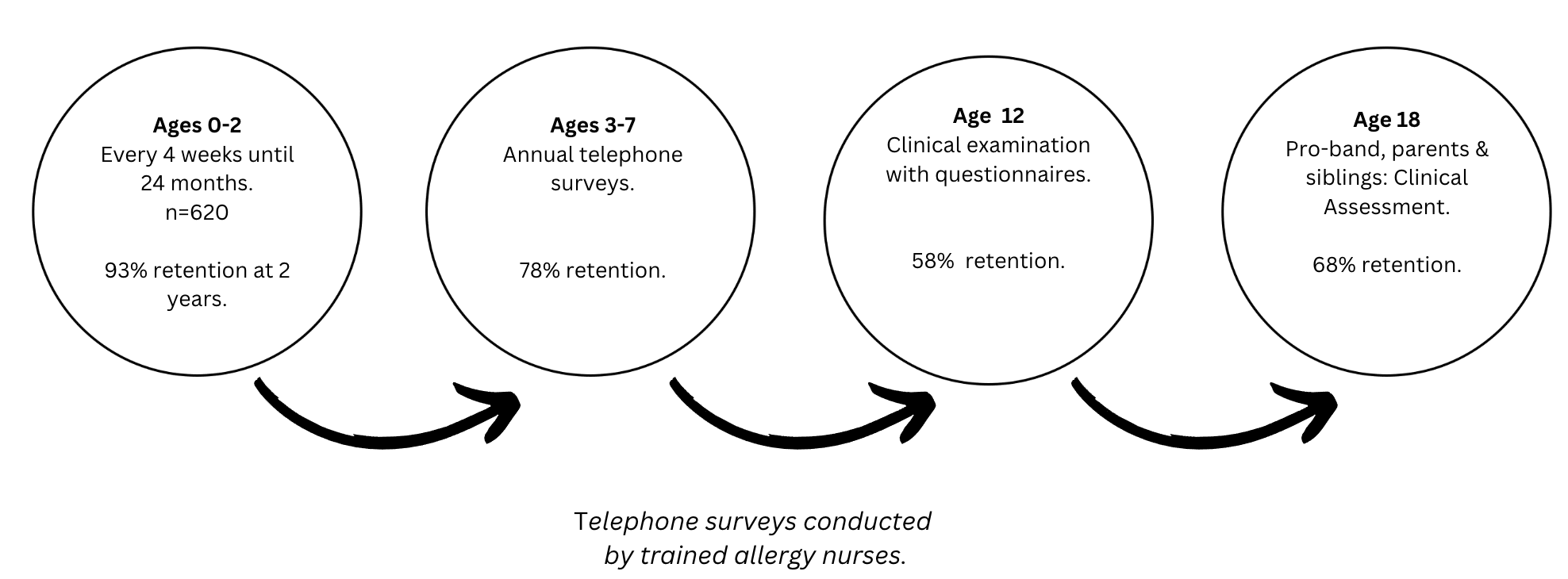


**khb**

**Supplementary Figure S2: Australian PBS DDI Rates 1990-1997 - Penicillins & Macrolides** ^[4]^

**
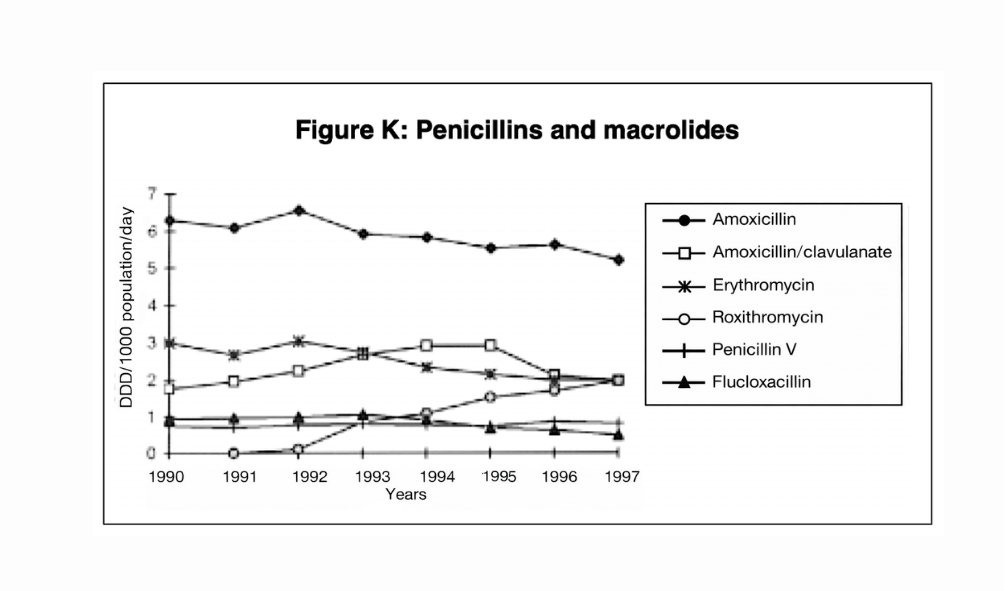
**

**Supplementary Figure S3: Directed Acyclic Graph**

**
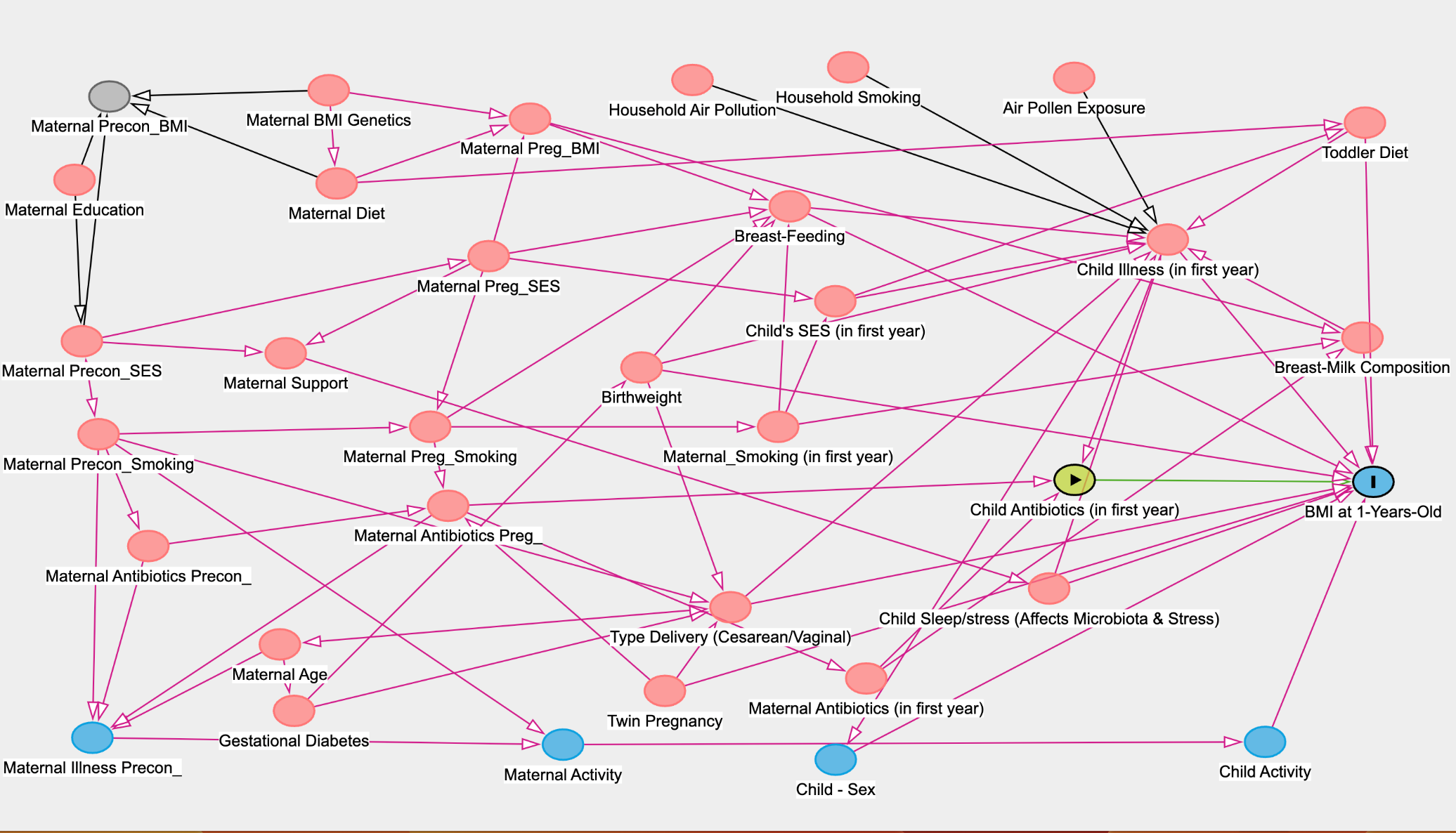
**

**Abbreviation:** BMI = Body Mass Index; Preg = Pregnancy; Precon= Preconception

**DAG: Minimal set of confounders for adjustment =** Delivery mode, maternal weight, maternal smoking preconception, maternal smoking pre-natal, parental smoking, maternal support, maternal antibiotics preconception, maternal antibiotics post-natal, birthweight, gestational diabetes, child sex, breast-feeding ≤ 3 months vs ≥3month, child asthma and allergy conditions, toddler diet, child activity/sedentary behaviour & child SES and child Illness in first year (infection rate).

**Supplementary Figure S4: Antibiotic Exposure (days) - Pwcorr Statistics**


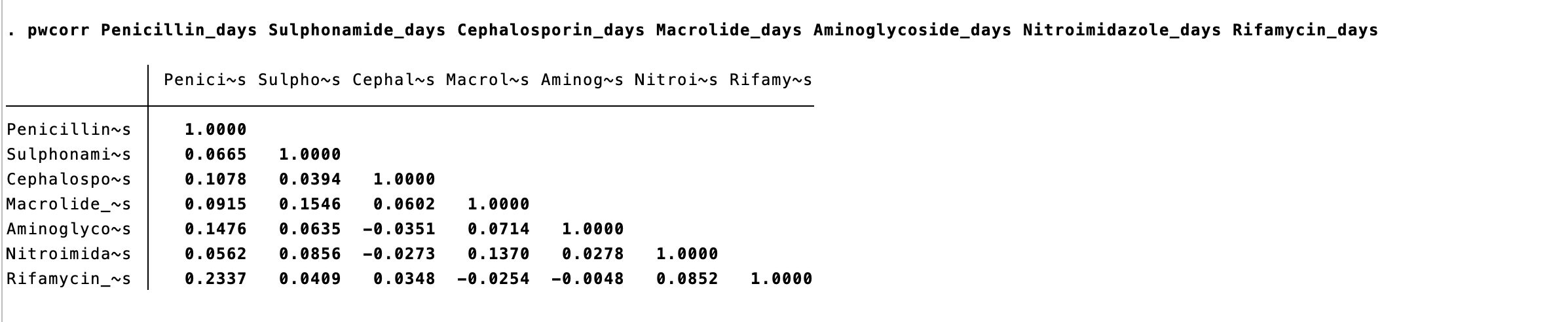


**Supplementary Figure S5: Multinomial Logistic Regression: Adjusted Odds Ratios (95% Confidence Intervals) - Dose-Dependent Antibiotic Use** $\boldsymbol{\leq}$ **2 Years of Age Across BMI Trajectory Groups (0-18 Years)**


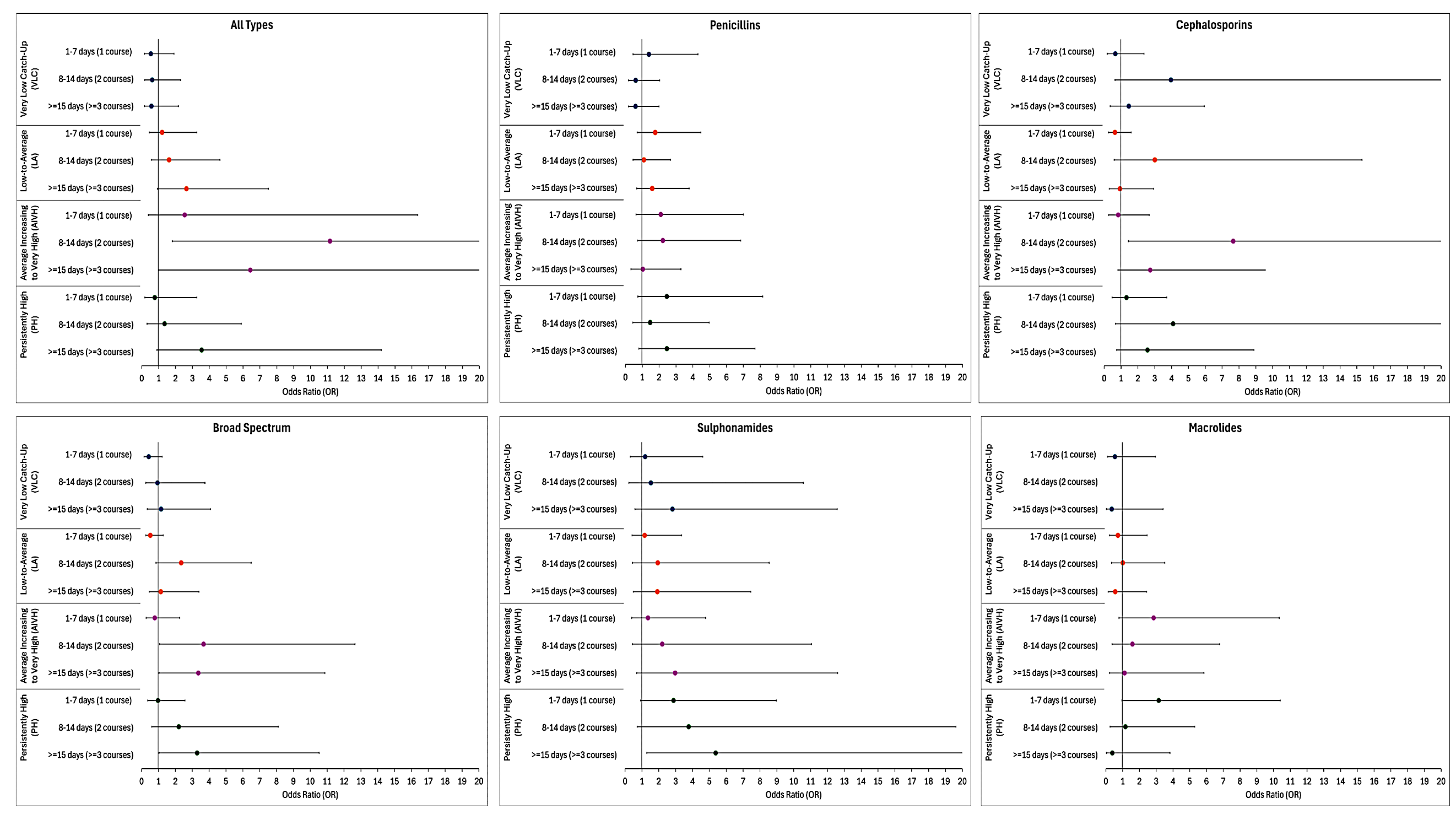


Adjusted for: gender, participant allergies, upper respiratory tract infection, bronchitis, otitis media, exclusive breast feeding, parental education, mother’s and father’s smoking history.

- Any antibiotic = Peniciilins, Sulphonamides, Cephalosporins, Macrolides & Nitroimidazoles.
- Broad Spectrum = Sulphonamides, Cephalosporins, Macrolides & Nitroimidazoles.

**References**

1. Lowe AJ, Lodge CJ, Allen KJ, Abramson MJ, Matheson MC, Thomas PS, et al. Cohort Profile: Melbourne Atopy Cohort study (MACS). Int J Epidemiol 2017; 46(1). Available from: https://pubmed.ncbi.nlm.nih.gov/27097746/

2. Gallagher C, Lambert K, Pirkis J, Abramson MJ, Barton C, Lodge CJ, et al. BMI trajectories from infancy to 18 years and mental health in emerging adulthood. J Affect Disord [Internet] 2025 [cited 2026 Jan 2];368:857–64. Available from: https://www.sciencedirect.com/science/article/abs/pii/S0165032724015891?via%3Dihub

3. Dai X, Dharmage SC, Abramson MJ, Erbas B, Bennett CM, Svanes C, et al. Early life acetaminophen exposure, glutathione S-transferase genes, and development of adolescent asthma in a high-risk birth cohort. Journal of Allergy and Clinical Immunology 2020;146(5):1035-1044.e12.

4. Commonwealth Department of Health and Family Services. Australian Statistics on Medicines 1997 [Internet]. 1998 [cited 2023 Nov 7]. Available from: https://www.pbs.gov.au/info/statistics/asm/asm-1997

**Supplementary Table S1**

| **Baseline Demographics of the MACS Cohort by BMI Trajectory Only (0–18 years) (n=434)** | | | | |
| --- | --- | --- | --- | --- |
| **Participant Characteristics (n=434)** | **% (n)** | **%(n)** | **%(n)** |  |
| Biological Sex* (n=434) | Boys (227) | Girls (207) |  |  |
| Exclusive Breastfeeding^‡^ (n=434) | 3 Months  45.39% (197) | > 3 Months  54.61% (237) |  |  |
| **Participant Yes/No Variables** | **Yes** | **No** |  |  |
| Asthma (n=434) | 55.76% (192) | 44.24% (242) |  |  |
| Hayfever (n=434) | 44.47% (193) | 55.53% (241) |  |  |
| Eczema (n=434) | 54.15% (235) | 45.85% (199) |  |  |
| All Allergies Variable (n=434) | 75.12% (326) | 24.88% (108) |  |  |
| **0-2 Years of Age Infection Episodes** | **Never** | **1-2 Episodes** | **3 Episodes** |  |
| Upper Respiratory Tract (URTI) ^‡^ (n=434) | 9.45% (41) | 70.74% (307) | 19.82% (86) |  |
| Bronchitis^‡^ (n=434) | 80.65% (350) | 14.75% (64) | 4.61% (20) |  |
| Otitis Media^‡^ (n=434) | 37.33% (162) | 28.11% (122) | 34.56% (150) |  |
| **Parental Characteristics** | | | |  |
| Delivery Mode^#^ (n=241) | Vaginal Birth 44.10% (191) | Cesarean (Elective & Emergency) 11.52% (50) |  |  |
| Mother Martial Status* (n=434) | Married or De facto  97.70% (424) | Single or Divorced  2.30% (10) |  |  |
| Mothers Education* (n=353) | Less than Secondary or Secondary Education 36.83% (130) | Tertiary Education 63.17% (223) |  |  |
| Fathers Education* (n=352) | Less than Secondary or Secondary Education  (137) | Tertiary Education (215) |  |  |
| Parent's Education (n=353) | Neither Parent Has Tertiary Education 26.91% (95) | One or Both Parents Has Tertiary Education 73.09% (258) |  |  |
| Mother's Smoking Status* (n=432) | Current or 3/12  6.25% (27) | Not Current, but 6/12  18.52% (80) | Never   75.23% (325) |  |
| Fathers Smoking Status* (n=428) | Current or 3/12 18.46% (79) | Not Current, but 6/12  13.79% (59) | Never  67.76% (290) |  |
| **Parent Yes/No Variables** | **Yes** | **No** |  |  |
| Mothers Smoking (first 4 weeks) ^‡^ (n=408) | 5.64% (23) | 94.36% (385) |  |  |
| Fathers Smoking (first 4 weeks) ^‡^ (n=408) | 14.95% (61) | 85.05% (347) |  |  |
| Mother Ever Smoked (combination of the two smoking variables) (pre-conception, In-utero & 4 weeks post pregnancy) (n=432) | 25.11% (109) | 74.42% (323) |  |  |
| Father Ever Smoked (combination of the two smoking variables) (pre-conception, In-utero & 4 weeks post pregnancy) (n=429) | 32.26% (140) | 66.58% (289) |  |  |
| **Primary Exposure Variable: Antibiotic Use** $\boldsymbol{\leq}$ **2 years of age (n=428) ^Þ^** | **Yes** | **No** |  |  |
| All Types | 82.71% (354) | 17.20% (74) |  |  |
| Broad-Spectrum (Sulphonamides, Cephalosporins, Macrolides, Nitroimidazoles) | 54.44% (233) | 45.56% (195) |  |  |
| Penicillins | 67.99% (297) | 30.60% (131) |  |  |
| Sulphonamides | 25.70% (110) | 74.29% (318) |  |  |
| Cephalosporins | 31.07% (133) | 68.92% (295) |  |  |
| Macrolides | 20.79% (89) | 79.21% (339) |  |  |
| Nitroimidazoles | 5.37%% (23) | 94.46% (405) |  |  |
| **^Þ^** Missing 6 Participants; *Measured at baseline; ^‡^Measured within the first 2 years of life.; ^#^Measured at 18 Year Follow Up; **^¥^**Measured at 25 Year Follow Up.  N.B: Discrepancy in the numbers of the different variables is attributed to missing data of specific variables | | | | |

| **Supplementary Table 2** | | | | | | |
| --- | --- | --- | --- | --- | --- | --- |
| **Demographics Across BMI Trajectory Groups (Ages 0-18 Years) Including Additional Smoking Variables for Participants Who Had Antibiotic Exposure Information** | | | | | | |
|  | **Very Low Catch-Up (VLC) (n=47, 11.0%)** | **Low-to-Average (LA) (n=146, 34.1%)** | **Stable Average (SA) (n=108, 25.2%)** Base outcome | **Average Increasing to Very High (AIVH)**  **(n=64, 15%)** | **Persistently High (PH) (n=63, 14.7%)** | ***p*-value** |
| **Participant Characteristics % (n); Total Sample Size (N=428) ^Þ^** | | | | | | |
| **Mother's Smoking Status*** |  |  |  |  |  | 0.961^b^ |
| Current or within 3/12 (n=325) | 4.3% (2) | 5.5% (8) | 7.2% (8) | 7.8% (5) | 6.3% (4) |  |
| Former smoker (ceased within the past 6 months) (n=80) | 14.9% (7) | 21.2% (31) | 16.2% (18) | 20.3% (13) | 17.2% (11) |  |
| Never (n=27) | 80.9% (38) | 73.3% (107) | 76.6% (85) | 71.9% (46) | 76.6% (49) |  |
| **Mother's Smoking (first 4 Weeks)** ^‡^  Yes (n=23) | 2.4% (1) | 2.8% (4) | 9.7% (10) | 6.5% (4) | 6.7% (4) | 0.176^b^ |
| **Father's Smoking Status*** |  |  |  |  |  | 0.221^a^ |
| Current or within 3/12 (n=290) | 14.9% (7) | 16.6% (24) | 15.3% (17) | 25.4% (16) | 24.2% (15) |  |
| Former smoker (ceased within the past 6 months) (n=59) | 17.0% (8) | 13.1% (19) | 9.9% (11) | 20.6% (13) | 12.9% (8) |  |
| Never (n=79) | 68.1% (32) | 70.4% (102) | 74.8% (83) | 54.0% (34) | 62.9% (39) |  |
| **Father's Smoking (first 4 Weeks)** ^‡^   Yes (n=61) | 12.2% (5) | 12.0% (17) | 15.5% (16) | 19.4% (12) | 18.3% (11) | 0.601^a^ |
| **Mother Ever Smoked** (pre-conception, In-utero & 4 weeks post pregnancy)  Ever (n=109) | 19.2% (9) | 26.7% (39) | 25.2% (28) | 28.1% (18) | 23.4% (15) | 0.829^a^ |
| **Father Ever Smoked** (pre-conception, In-utero & 4 weeks post pregnancy) Ever (n=140) | 31.9% (15) | 30.1% (44) | 26.1% (29) | 46.0% (29) | 37.1% (23) | 0.082^a^ |
| **^Þ^** Missing 6 Participants; *Measured at baseline; ^‡^Measured within the first 2 years of life; ^#^Measured at 18 Year Follow Up; **^a^***p*-value derived from chi-square test, ^b^ *p*-value derived from Fisher’s exact test. | | | | | | |

|  | | | | | |
| --- | --- | --- | --- | --- | --- |
| **Multinomial Logistic Regression Adjusted Odds Ratios (95% CI) - Antibiotic Use** $\boldsymbol{\leq}$ **2 Years of Age Across BMI Trajectory Groups (0-18 Years)** | | | | | |
|  | **Very Low Catch-Up (VLC) (n=47)** | **Low-to-Average (LA) (n=146)** | **Stable Average (SA) (n=108)** Base outcome | **Average Increasing to Very High (AIVH) (n=64)** | **Persistently High (PH) (n=63)** |
| **Antibiotic Class (n=yes) 2 Years of Age** **^Þ ‡^ (aOR, 95% CI, p-value); Total Sample Size (n=428)** | | | | | |
| **Any antibiotic** (n=354) | 0.56 (0.18, 1.69) p=0.304 (n=36) | 1.66 (0.69, 3.97) p=0.254 (n=116) | (n=86) | 5.19 (0.97, 27.80) p=0.054 (n=60) | 1.56 (0.46, 5.28) p=0.475 (n=56) |
| **Broad-Spectrum** (Sulphanomides, Cephalosporins, Macrolides & Nitroimidazoles) (n=242) | 0.63 (0.25, 1.54) p=0.317 (n=24) | 0.91 (0.47, 1.77) p=0.791 (n=75) | (n=51) | 1.87 (0.77, 4.56) p=0.165  (n=23) | 1.69 (0.71, 3.98) p=0.230 (n=22) |
| **Penicillins** (n=297) | 0.83 (0.39, 2.08) p=0.693 (n=32) | 1.45 (0.72, 2.95) p=0.295 (n=93) | (n=72) | 1.67 (0.64, 4.82) p=0.146 (n=50) | 2.21 (0.79, 5.65) p=0.062 (n=50) |
| **Sulphonamides** (n=139) | 1.65 (0.61, 4.48) p=0.321 (n=13) | 1.52 (0.68, 3.37) p=0.301 (n=31) | (n=21) | 1.95 (0.79, 5.38) p=0.101 (n=19) | 3.69 (1.57, 9.03) p=0.004 (n=26) |
| **Cephalosporins** (n=176) | 1.21 (0.48, 3.04) p=0.682 (n=16) | 0.95 (0.46,1.96) p=0.897 (n=36) | (n=30) | 2.04 (0.88,4.73) p=0.095 (n=28) | 1.94 (0.84, 4.48) p=0.118 (n=23) |
| **Macrolides** (n=124) | 0.30 (0.08, 1.15) p=0.080 (n=4) | 0.81 (0.36, 1.78) p=0.594 (n=26) | (n=25) | 1.87 (0.74,4.77) p=0.185 (n=16) | 1.65 (0.66, 4.16) p=0.281 (n=18) |
| ^Þ^Missing 6 Participants; ^‡^Measured within the first 2 years of life; ^€^Adjusted for gender, participant allergies, upper respiratory tract infection, bronchitis, otitis media, exclusive breast feeding, parental education, mother’s and father’s smoking history. | | | | | |

**Supplementary Table S3**

**Supplementary Table S4**

| **Sensitivity Analysis: Multinomial Logistic Regression: Participant Confounder Effect Size Odds Ratios (95% CIs) - All Antibiotic Type Antibiotic Type Exposure Variable ≤ 2 Years of Age Across BMI Trajectories (Age 0-18 Years)** | | | | | | |
| --- | --- | --- | --- | --- | --- | --- |
| **Variable Total n = 428** | **p-value (Distribution)** | **Very Low Catch-Up (n=47)** | **Low-to-Average (n= 145-146)** | **Stable Average (n=108-112) Base outcome** | **Average Increasing to Very High (n=64)** | **Persistently High (n=62-63)** |
| All-Types Antibiotic  Unadjusted ORs (95%)  (n=yes) | 0.026^b^ | 0.84 (0.37, 1.90)  p=0.672  (n=36) | 0.99 (0.53, 1.83)  p=0.972  (n=116) | Base Outcome (n=86) | 3.84 (1.26, 11.704)  p=0.018  (n=60) | 2.05 (0.82, 5.11)  p=0.125  (n=56) |
| **Participant Confounders** | | | | | | |
| Sex (Male vs Female) | 0.660^a^ | 0.86 (0.38, 1.98) p= 0.725 | 0.99 (0.53, 1.84) p=0.984 |  | 3.82 (1.25, 11.66) p=0.019 | 2.05 (0.82, 5.14) p=0.123 |
| Exclusive Breast Feeding (<3months vs > 3 months) | 0.005^a^ | 0.81 (0.35, 1.86) p=0.631 | 0.98 (0.52, 1.82) p=0.952 e |  | 3.66 (1.19, 11.23) p=0.024 | 1.97 (0.79, 4.96) p=0.145 |
| **Allergy Variables** | | | | | | |
| All Allergies (Yes/No) | 0.133^a^ | 0.80 (0.35, 1.85) p=0.606 | 0.88 (0.46, 1.65) p=0.692 |  | 3.77 (1.23, 11.59) p=0.020 | 1.96 (0.78, 4.92) p=0.153 |
| Separate entities added: Asthma, Eczema & Hay-fever  (See Table 2 for n) | N/A | 0.61 (0.20, 1.87) p=0.385 | 1.39 (0.57, 3.39) p=0.465 |  | 5.91 (1.09, 32,13) p=0.039 | 2.02 (0.56, 7.37) p=0.284 |
| Ever Asthma (Yes/No) | 0.323^a^ | 0.85 (0.37, 1.95) p=0.704 | 0.91 (0.49, 1.70) p=0.770 |  | 3.64 (1.18, 11.17) p=0.024 | 2.03 (0.81, 5.11) p=0.131 |
| Ever Eczema (Yes/No) | 0.293^a^ | 0.82 (0.36, 1.87) p=0.640 | 0.97 (0.52, 1.80) p=0.931 |  | 3.93 (1.29, 12.02) p=0.016 | 2.00 (0.80, 4.99) p=0.140 |
| Ever Hayfever (Yes/No) | 0.415^a^ | 0.76 (0.33, 1.76) p=0.523 | 0.87 (0.46, 1.63) p=0.667 |  | 3.70 (1.20, 11.38) p=0.022 | 1.83 (0.72, 4.63) p=0.201 |
| **Infection (Yes/No)** | | | | | | |
| Upper Respiratory Tract Infection | Stata Unable to Detect | 0.79 (0.33, 1.90) p=0.602 | 1.18 (0.61, 2.30) p=0.620 |  | 3.66 (1.16, 11.53) p=0.026 | 2.04 (0.77, 5.37) p=0.150 |
| Bronchitis | 0.807^b^ | 0.86 (0.38, 1.96) p=0.721 | 1.01 (0.54, 1.87) p=0.981 |  | 3.84 (1.25, 11.75) p=0.018 | 2.07 (0.82, 5.19) p=0.119 |
| Otitis Media | 0.078^b^ | 0.93 (0.36, 2.37) p=0.880 | 1.35 (0.67, 2.72) p=0.399 |  | 4.68 (1.42, 15.42) p=0.011 | 2.09 (0.77, 5.73) p=0.149 |
|  | | | | | | |

| **Sensitivity Analysis: Multinomial Logistic Regression: Parental Confounder Effect Size Odds Ratios (95% CI) - All Antibiotic Type Exposure Variable ≤ 2 Years of Age Across BMI Trajectories (Age 0-18 Years)** | | | | | | |
| --- | --- | --- | --- | --- | --- | --- |
| **Variable Total n = 428** | **p-value (Distribution)** | **Very Low Catch-Up (n=47)** | **Low-to-Average (n= 145-146)** | **Stable Average (n=108-112) Base outcome** | **Average Increasing to Very High (n=64)** | **Persistently High (n=62-63)** |
| All-Types Antibiotic  Unadjusted ORs (95%)  (n=yes) | 0.026^b^ | 0.84 (0.37, 1.90)  p=0.672  (n=36) | 0.99 (0.53, 1.83)  p=0.972  (n=116) | Base Outcome (n=86) | 3.84 (1.26, 11.704)  p=0.018  (n=60) | 2.05 (0.82, 5.11)  p=0.125  (n=56) |
| **Parent Confounders** | | | | | | |
| Mother Ever Smoke (Yes/No) | 0.829^a^ | 0.78 (0.34, 1.79) p=0.551 | 0.99 (0.53, 1.85)  p=0.983 |  | 3.74 (1.21, 11.46) p=0.021 | 1.94 (0.77, 4.87) p=0.083 |
| Paternal Ever Smoke (Yes/No) | 0.082^a^ | 0.78 (0.34, 1.79)  p=0.563 | 0.96 (0.51, 1.81) p=0.906 |  | 3.397 (1.10, 10.40)  p=0.034 | 1.82 (0.72, 4.59)  p=0.201 |
| Parent Education  (Either Tertiary Vs No Tertiary) | 0.304^a^ | 0.82 (0.34, 1.98) p=0.661 | 1.18 (0.59, 2.36) p=0.636 |  | 7.05 (1.57, 31.70) p=0.011 | 2.00 (0.74, 5.42) p=0.172 |
|  | | | | | | |

**Supplementary Table S5**
